# Characterizing health care provider knowledge: Evidence from HIV services in Kenya, Rwanda, South Africa, and Zambia

**DOI:** 10.1371/journal.pone.0260571

**Published:** 2021-12-02

**Authors:** Carlos Pineda-Antunez, David Contreras-Loya, Alejandra Rodriguez-Atristain, Marjorie Opuni, Sergio Bautista-Arredondo

**Affiliations:** 1 National Institute of Public Health (INSP), Division of Health Economics and Health Systems Innovations, Cuernavaca, Mexico; 2 School of Public Health, University of California, Berkeley, Berkeley, California, United States of America; 3 Independent consultant, Geneva, Switzerland; Universidad Loyola Andalucia Cordoba, SPAIN

## Abstract

**Background:**

Identifying approaches to improve levels of health care provider knowledge in resource-poor settings is critical. We assessed level of provider knowledge for HIV testing and counseling (HTC), prevention of mother-to-child transmission (PMTCT), and voluntary medical male circumcision (VMMC). We also explored the association between HTC, PMTCT, and VMMC provider knowledge and provider and facility characteristics.

**Methods:**

We used data collected in 2012 and 2013. Vignettes were administered to physicians, nurses, and counselors in facilities in Kenya (66), Rwanda (67), South Africa (57), and Zambia (58). The analytic sample consisted of providers of HTC (755), PMTCT (709), and VMMC (332). HTC, PMTCT, and VMMC provider knowledge scores were constructed using item response theory (IRT). We used GLM regressions to examine associations between provider knowledge and provider and facility characteristics focusing on average patient load, provider years in position, provider working in another facility, senior staff in facility, program age, proportion of intervention exclusive staff, person-days of training in facility, and management score. We estimated three models: Model 1 estimated standard errors without clustering, Model 2 estimated robust standard errors, and Model 3 estimated standard errors clustering by facility.

**Results:**

The mean knowledge score was 36 for all three interventions. In Model 1, we found that provider knowledge scores were higher among providers in facilities with senior staff and among providers in facilities with higher proportions of intervention exclusive staff. We also found negative relationships between the outcome and provider years in position, average program age, provider working in another facility, person-days of training, and management score. In Model 3, only the coefficients for provider years in position, average program age, and management score remained statistically significant at conventional levels.

**Conclusions:**

HTC, PMTCT, and VMMC provider knowledge was low in Kenya, Rwanda, South Africa, and Zambia. Our study suggests that unobservable organizational factors may facilitate communication, learning, and knowledge. On the one hand, our study shows that the presence of senior staff and staff dedication may enable knowledge acquisition. On the other hand, our study provides a note of caution on the potential knowledge depreciation correlated with the time staff spend in a position and program age.

## Introduction

Ending the AIDS epidemic by 2030 is a target of the Sustainable Development Goals (SDGs) [1]. Accompanying this objective are the Joint United Nations Programme on HIV/AIDS (UNAIDS) goal of reducing the number of new HIV infections by 90% between 2010 and 2030, and its 95-95-95 target which proposes that by 2030, 95% of people living with HIV should be diagnosed, of whom 95% should be on treatment, of whom 95% should be virally suppressed [2]. The world is not on track to reach these objectives despite remarkable progress in the global response to HIV in recent years [3]. Experts highlight “suboptimal or inappropriate policies, lack of funding, [and] limited or misdirected implementation of available strategies and tools” to explain why progress has been slower than foreseen [4]. According to the World Health Organization (WHO), gaps in service access and quality span the cascade of HIV services, and countries will reach HIV targets only if they substantially improve quality of care [5].

Improving quality of care in low- and middle-income countries (LMICs) has become a global priority encompassing all health care services [6–8], with quality of care defined as “the degree to which health care services for individuals and populations increase the likelihood of desired outcomes and are consistent with current professional knowledge [7].” There is a growing literature describing the substantial deficiencies that exist in quality of care in resource-poor settings (e.g. [9–16]) and an increasing number of studies documenting poor health care provider performance in LMICs [6, 7] and low levels of provider knowledge [17–28]. Several factors have been identified as barriers to provider adherence to clinical guidelines and implementation of evidence-based practices [7]. At the organizational level, these factors include lack of supervision and mentorship, poor communication, information sharing, and collaboration within individual health care facilities [7, 29]. At the provider level, substandard pre-service training and continuing education have been highlighted as significant barriers [6, 29]. In addition, experts have shown that management practices and management capacity in LMIC health care facilities are poor and improving these is key to improving quality of care in these settings [6].

This paper contributes to the body of work on provider knowledge in resource-limited settings. Following Donabedian, we conceptualized provider knowledge as a dimension of structural quality and a necessary but insufficient condition for adequate process quality [30–32]. We assumed that provider knowledge can accumulate over time, but that it can also depreciate [33, 34]. Some explanations for knowledge depreciation include the evolution of knowledge and the time it takes to diffuse new guidelines and evidence [35], lack of motivation among staff to acquire knowledge and skills [36], and staff turnover and organizational inertia in facilities [37]. We also assumed that in addition to learning through education and training, individuals embedded in organizations also learn from knowledge reservoirs (e.g. treatment guidelines), tools, learning by doing, social networks [38], and transactive memory systems that develop when people in groups develop ways of assigning responsibility for information based on shared understanding of one another’s knowledge [39, 40].

In this analysis, we focused on provider knowledge for three outpatient HIV services: HIV testing and counseling (HTC), prevention of mother-to-child transmission (PMTCT), and voluntary medical male circumcision (VMMC). We used vignettes to assess knowledge of HTC, PMTCT, and VMMC guidelines among HIV service providers in health care facilities in four countries in sub-Saharan Africa: Kenya, Rwanda, South Africa, and Zambia. We also explored the association between HTC, PMTCT, and VMMC provider knowledge and provider and facility characteristics drawn from literature in organizational psychology, economics, management, and production research.

## Methods

### Study design and sample

We used data collected as part of the “Optimizing the Response in Prevention: HIV Efficiency in Africa” (ORPHEA) study, which was a cross-sectional, micro-costing study that collected year-long data in health care facilities in Kenya, Rwanda, South Africa, and Zambia. We collected data in 2012 in Kenya, South Africa, and Zambia and in 2013 in Rwanda. The ethical review boards of the following institutions approved the study: National Institute of Public Health, Mexico; Kenyatta National Hospital and University of Nairobi; Northeastern University; Rwanda Biomedical Center; University of the Witwatersrand; and University of Zambia. We have published elsewhere detailed descriptions of the sampling and data collection methods used [41–44] and briefly summarize them here. In each country, we used multistage sampling to first select sub-national areas and then randomly select facilities providing at least one of the following outpatient HIV services: HTC, PMTCT, and VMMC (see S1 Table for descriptions of the HTC, PMTCT, and VMMC services implemented in Kenya, Rwanda, South Africa, and Zambia at the time of the ORPHEA study). We enumerated facilities in selected sub-national areas along with information on the services of interest stratified by ownership and level of service provision. We randomly sampled facilities within these strata, using probability proportional to size, giving preference to sites providing more than one service of interest. In sampled facilities, we randomly selected up to five providers who had contact with HTC, PMTCT, and VMMC patients to take part in the vignette-based interviews described below. The ORPHEA study assembled the detailed input and output data required to estimate the annual cost of service provision of HTC, PMTCT, and VMMC per patient [41–44]. In addition, the study also collected data on: provider knowledge; provider attributes (e.g. age, cadre, education); and facility characteristics (e.g. type, ownership, management practices). The sub-sample we used in this paper is limited to those providers and facilities with complete information on provider and facility characteristics.

### Provider knowledge measure

We administered medical vignettes for the three outpatient HIV services considered in the ORPHEA study: HTC, PMTCT, and VMMC. Previous studies have shown that vignettes reliably measure the medical knowledge of health care providers [18, 26, 45–48]. ORPHEA study vignettes were paper-based hypothetical scenarios describing HTC, PMTCT, and VMMC patients. The HTC vignette described a 32-year-old married woman testing for HIV for the first time; the PMTCT vignette represented a pregnant 26-year-old married woman living with HIV attending her second antenatal care visit; and the VMMC vignette described an HIV-negative 26-year-old man attending pre- and post-operative counselling. We asked respondents a series of questions about the activities they would carry out with the patients in these hypothetical scenarios, reproducing the actions they would undertake with actual patients. Caregivers sampled included physicians, nurses, and counselors interacting with HTC, PMTCT, and VMMC patients. Providers of HTC, PMTCT, and VMMC services were each assessed using the vignette pertaining to their area of work. We evaluated health care workers for each of the three interventions using the same vignette irrespective of professional category. National and World Health Organization HTC, PMTCT, and VMMC guidelines in place at the time of the study were used to assess the responses provided. For this study, we retained only those items that conformed to global guidance and were consistent across the four countries. We evaluated 112 items for HTC, 89 items for PMTCT, and 76 items for VMMC. We scored all items dichotomously, with a value of 1 if the provider mentioned the item and 0 if the provider did not (see S2 Table for average percent of items correctly identified by intervention, provider cadre, and country).

We constructed three provider knowledge indices—one for HTC, one for PMTCT, and another for VMMC—with each index combining the information collected from all HTC, PMTCT, and VMMC providers across the four countries. To construct the three indices, we used item response theory (IRT) [9, 18, 49]. IRT is a collection of models in which an individual’s level of a latent trait (i.e. knowledge) depends on their responses to a series of items and on the characteristics of those items [50, 51]. The IRT models we estimated considered two-item characteristics: item difficulty (level of item difficulty given provider knowledge) and item discrimination (capacity of the item to distinguish between providers able to respond correctly from those who were not). We applied a second order IRT model which allows items to vary by both difficulty and discrimination. To estimate the expected knowledge score for each provider, we summed the probabilities of providing the correct answer for each of the items. We rescaled each knowledge score to range from 0 to 100, where the two extremes represent the lowest and the highest scores observed in our sample. We tested the internal consistency of the knowledge scores using Cronbach’s Alpha (α = 0.95 for HTC, 0.92 for PMTCT, and 0.94 for VMMC). To further validate the estimated HTC, PMTCT, and VMMC knowledge scores, we split the samples into tertiles (low, medium, and high) and compared the proportion of correct answers to the ten most difficult items for each intervention across the three knowledge levels. High knowledge providers were more likely to respond correctly to each of the ten most difficult HTC, PMTCT, and VMMC items, while low knowledge providers were less likely to do so (S1 Fig).

### Provider and facility characteristics of interest

In our exploration of factors associated with HTC, PMTCT, and VMMC provider knowledge, we focused on provider and facility characteristics drawn from literature in organizational psychology, economics, management, and production research, while adjusting for factors identified as predictors of health care worker performance in previous work [9, 21, 52–59]. Our main variables of interest were: average patient load (average number of patients seen per week), provider years in position (number of years a provider has been in current position), provider working in another facility (whether the provider also works in another facility), senior staff (whether senior providers work in a facility), program age (number of years that a facility has been implementing HTC, PMTCT, and/or VMMC), proportion of exclusive staff (proportion of staff in a facility that works exclusively on one HIV intervention), training person-days (number of person-days of training provided to staff in a facility), and management score. The concepts that these variables aim to operationalize are shown in Table 1. We constructed both the senior staff variable and the management score. For the senior staff variable, we used principal component analysis (PCA) to construct a seniority score for all providers in the sample using years of education, years of experience, years in position, and cadre. We then grouped the seniority scores for each country into tertiles. Providers in the highest tertile were considered senior staff. The senior staff variable is a facility-level dummy variable, that is 1 if the facility has at least one senior provider and 0 otherwise. We created the management score based on information on facility-level management practices collected in the ORPHEA study through in-person interviews with managers responsible for HTC, PMTCT, and VMMC services. The interview questions were based on the World Management Survey (WMS) developed for manufacturing firms [60] and applied to health care facilities [54–57, 59]. We adapted questions to capture information relevant to health care facilities delivering HIV services [44, 61]. Our approach captured information on management practices in four domains: operations management, performance monitoring, people management, and community engagement. We constructed additive scores for each of these domains based on 68 management practice items (23 items for operations management, 15 for performance monitoring, 19 for people management, and 11 for community engagement) (See S3 Table for the list of management practice items). We then constructed an overall management score, including the 68 items together. We tested the internal consistency of the management scores using Cronbach’s Alpha (α = 0.85). The scores were standardized on a 0 to 100 scale. Multiple imputation was used to impute less than 5% of the management items that had missing values.

**Table 1:** Provider and facility characteristics of interest.

Concept	Variable name	Definition
Workload	Average patient load (weekly)	A measure of the intensity with which providers learn by doing. We hypothesized that providers with higher knowledge have higher workloads.
Seniority	Senior staff	The presence of more experienced and educated staff is a proxy for champions, who exert influence in the organization through higher status, intrinsic motivation, and commitment to implementing improvements.
Years in position	Provider years in position	A measure of knowledge depreciation at the individual level. Ceteris paribus, staff with longer tenure have lower levels of knowledge.
Program age	Average program age (years)	A measure of knowledge depreciation at the facility level. Ceteris paribus, staff in older HIV units have lower levels of knowledge.
Staff dedication/specialization	Provider works in another facility	Whether the provider also works in another facility is a measure of staff dedication. We hypothesized that providers who work in another facility have lower knowledge scores.
	Average % of exclusive staff for HIV	The proportion of clinical staff that is exclusively devoted to HIV prevention interventions is a measure of the degree of specialization of an HIV care unit.
Training	Average number of person-days of training	Actions undertaken at the organizational and macro-organizational level to develop and enhance the knowledge, skills, and competencies of employees.
Management	Management score	A set of coordinated activities oriented towards the achievement of organizational goals by leveraging human and technological resources.

### Data analysis

We estimated the mean, median, and standard deviation (SD) of the HTC, PMTCT, and VMMC provider knowledge scores and for the provider and facility characteristics. To explore the heterogeneity in HTC, PMTCT, and VMMC provider knowledge scores within countries, we mapped the distributions by country. To assess the heterogeneity across countries, we mapped the country distributions of provider knowledge scores for the three interventions.

We modeled the association between HTC, PMTCT, and VMMC provider knowledge and provider and facility attributes. We used generalized linear model (GLM) estimation—a maximum likelihood generalization of the ordinary linear regression approach. We specified a Poisson family distribution and the identity as the link function using Akaike and Bayes criteria to identify the model with the best goodness of fit.

The models were estimated as:

$$y_{ij}=\beta_{0}+\beta_{1}PL_{ij}+\;\beta_{2}YP_{ij}+\beta_{3}OF_{ij}+\gamma_{1}SS_{j}+\gamma_{2}PA_{j}+\gamma_{3}PE_{j}+\;\gamma_{3}TP_{j}+\gamma_{3}MS_{j}+X_{ij}^{\prime }\delta +\varepsilon_{ij}$$

where *y*_*ij*_ is the provider knowledge score of provider *i* in facility *j*, *PL*_*ij*_ is the provider patient load in log, *YP*_*ij*_ is the number of years a provider has been in current position, *OF*_*ij*_ indicates whether the provider worked in another facility, *SS*_*j*_ indicates whether the facility had at least one senior staff member, *PA*_*j*_ denotes program age, *PE*_*j*_ is the proportion of intervention exclusive staff, *TP*_*j*_ is the number of training person-days, *MS*_*j*_ is the management score. *β* and *γ* are the regression coefficients for provider and facility characteristics respectively. *X* is the vector of control variables (provider age, provider years of education, provider years of experience, provider cadre, intervention, urbanicity, facility type, facility ownership, number of annual patients, and country) and *δ* represents the regression coefficients for the control variables (see S4 Table for variable definitions and descriptions). We included interactions for country with cadre, and country with intervention. We report models with robust and clustered standard errors to account for the heteroskedasticity of the error term *ε*_*ij*_ and unobserved intra-facility heterogeneity without explicitly modelling intra- and inter-cluster correlation. The clustered specification assumes that regression model errors are independent across facilities but correlated within facilities [62, 63]. We examined the Variance Inflation Factor (VIF) to assess multicollinearity.

For four of our variables of interest that were continuous (provider years in position, program age, proportion of exclusive staff, and management score), we computed the expected knowledge at different percentiles of the variables of interest (using the model with non-clustered standard errors).

## Results

The analytic sample comprised 755 providers of HTC, 709 providers of PMTCT, and 332 providers of VMMC (Table 2). These providers came from 66 facilities in Kenya, 67 facilities in Rwanda, 57 facilities in South Africa, and 58 facilities in Zambia (Table 3). The average knowledge score was 36 for each of the three interventions.

**Table 2:** Provider-level characteristics, by HIV prevention intervention.

	HTC (n = 755)			PMTCT (n = 709)			VMMC (n = 332)		
	Mean	Median	SD	Mean	Median	SD	Mean	Median	SD
Knowledge score	36.3	33.6	16.4	36.4	35.5	14.5	36.3	34.6	18.0
Physician, %	5.03	-	21.9	5.50	-	22.8	20.5	-	40.4
Nurse, %	41.1	-	49.2	77.7	-	41.7	59.3	-	49.2
Counsellor, %	53.9	-	49.9	16.8	-	37.4	20.2	-	40.2
Provider age (years)	36.8	35.0	9.30	38.5	37.0	9.80	35.7	34.0	8.90
Provider years of education	14.3	14.0	2.60	15.1	15.0	2.70	15.3	15.0	2.70
Provider years of experience	13.3	11.0	7.20	15.2	12.0	8.80	12.9	11.0	6.60
Provider years in position	4.27	3.00	4.20	4.78	3.00	5.30	3.8	3.00	4.10
Average patient load (weekly)	124	75.0	115	155	125	118	129	90.0	122
Provider works in another facility, %	2.91	-	16.8	2.68	-	16.2	9.64	-	29.6

Notes: HTC, HIV testing and counseling; PMTCT, Prevention of Mother-to-Child Transmission; VMMC, Voluntary Medical Male Circumcision; The physician category includes: physicians, medical officers, clinical officers, and heads of service.

**Table 3:** Facility-level characteristics, by country.

	Kenyan (n = 66)			Rwanda (n = 67)			South Africa (n = 57)			Zambia (n = 58)		
	Mean	Median	SD	Mean	Median	SD	Mean	Median	SD	Mean	Median	SD
Private facilities, %	37.7	-	48.7	38.7	-	48.8	30.1	-	46.0	15.4	-	36.3
Hospitals, %	59.1	-	49.4	7.36	-	26.2	50.7	-	50.2	16.2	-	37.0
Rural facilities, %	29.5	-	45.8	67.3	-	47.1	64.0	-	48.2	59.4	-	49.4
Total number of annual patients (thousands)	2.45	1.52	3.37	2.69	1.21	4.45	2.88	1.96	4.91	1.08	0.83	1.13
Average program age (years)	7.64	7.17	5.14	5.83	5.92	4.31	6.60	7.25	4.06	7.59	7.13	5.41
Average % of exclusive staff for HIV	27.0	-	21.9	8.80	-	12.2	25.8	-	18.3	17.0	-	17.9
Average number of person-days of training	25.6	15.0	35.7	4.32	4.11	1.89	2.09	2.00	1.47	6.22	5.00	5.97
Senior staff, %	26.8	-	44.5	30.7	-	46.3	33.6	-	47.4	29.4	-	45.7
Management score	47.2	48.6	23.3	73.3	77.1	17.1	53.0	54.3	18.5	50.8	57.1	23.2

Notes: HTC, HIV testing and counseling; PMTCT, Prevention of Mother-to-Child Transmission; VMMC, Voluntary Medical Male Circumcision. Private facilities include for-profit, nonprofit, and faith-based health care facilities; average program age is the number of years HTC, PMTCT, and VMMC programs have been active at the facility; senior staff indicates whether at least one staff member ranked in the uppermost tertile of the seniority score.

**Fig 1:**
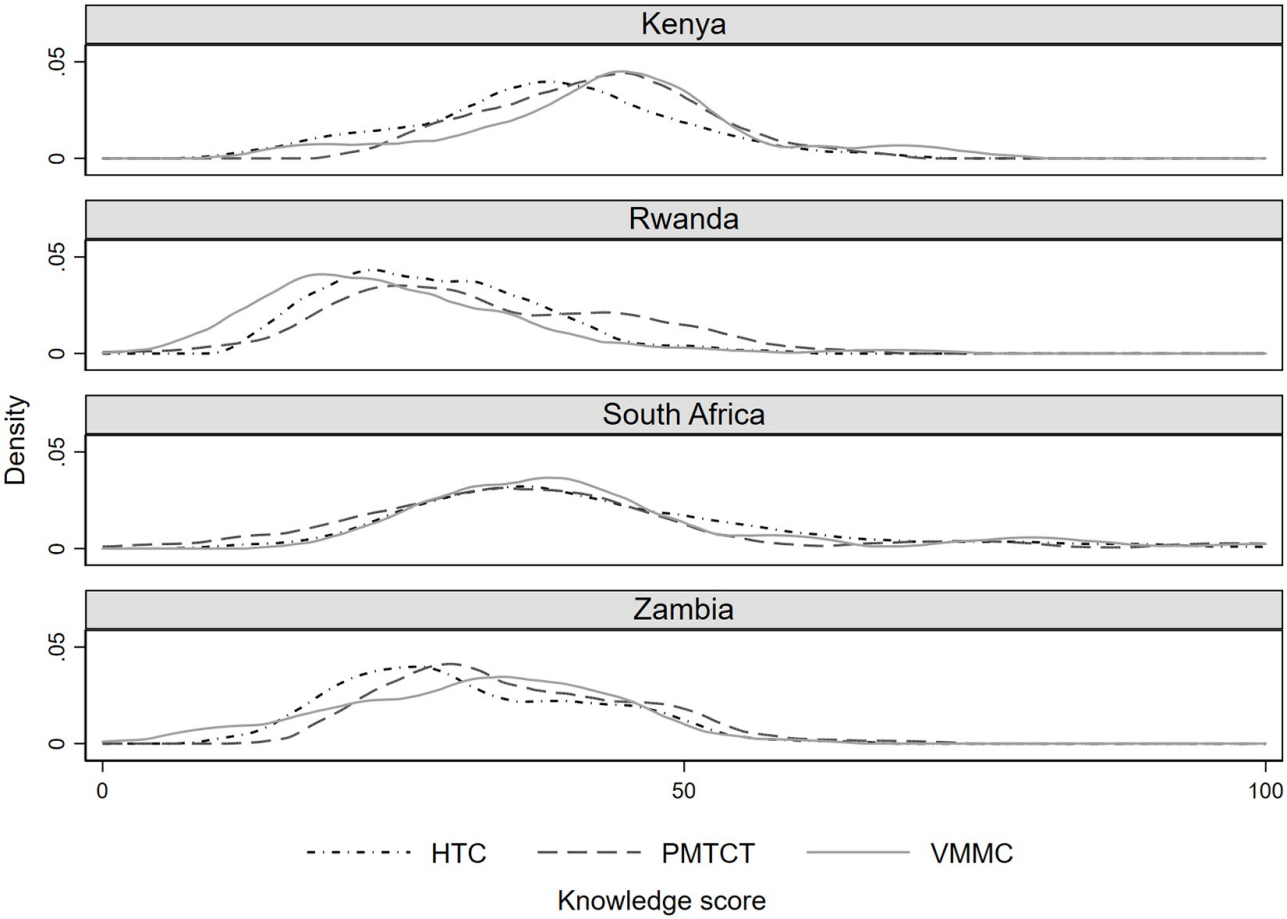
HTC, PMTCT, and VMMC provider knowledge scores in Kenya, Rwanda, South Africa, and Zambia. Notes: HTC, HIV testing and counseling; PMTCT, Prevention of Mother-to-Child Transmission; VMMC, Voluntary Medical Male Circumcision.

**Fig 2:**
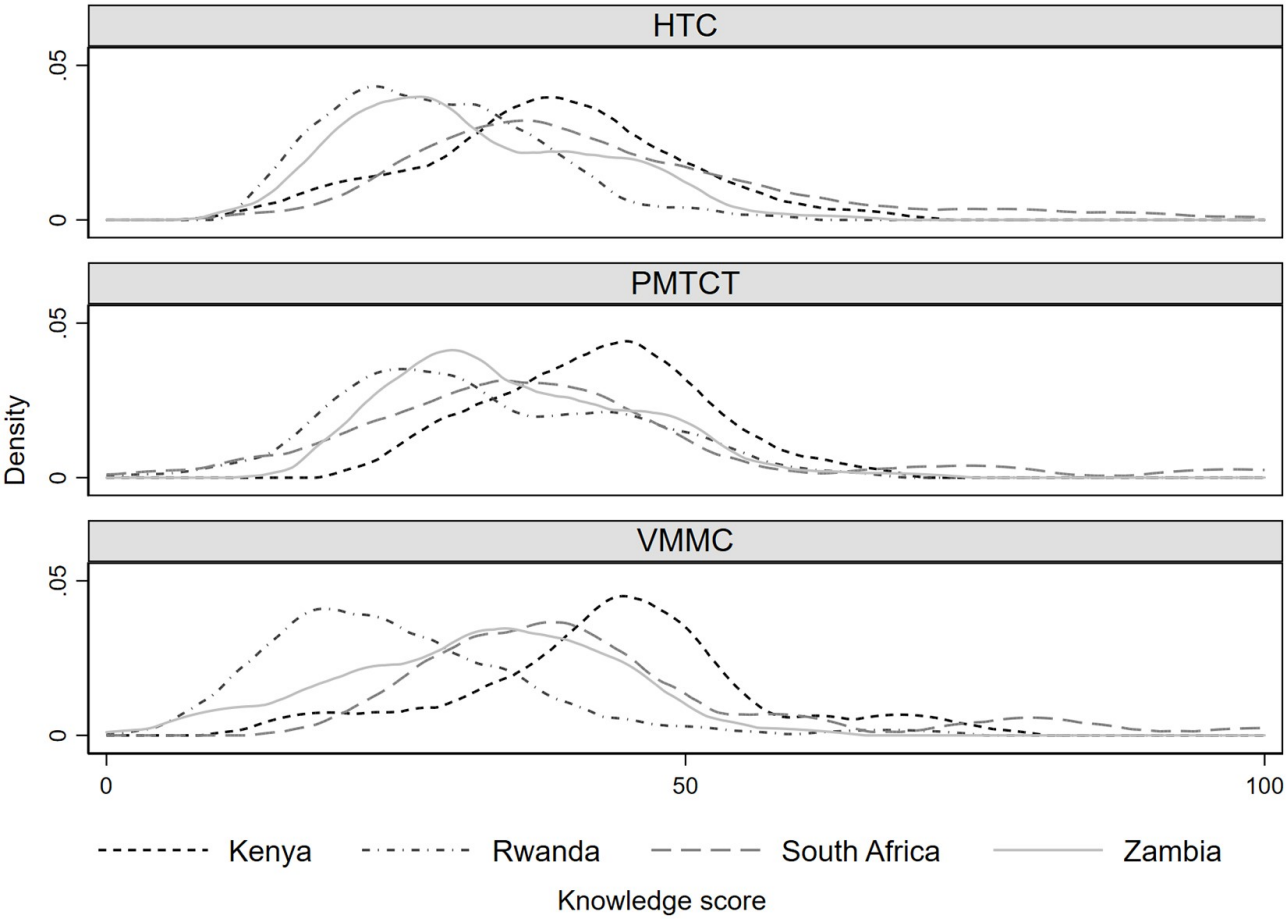
Provider knowledge scores in Kenya, Rwanda, South Africa, and Zambia, by intervention. Notes: HTC, HIV testing and counseling; PMTCT, Prevention of Mother-to-Child Transmission; VMMC, Voluntary Medical Male Circumcision.

Overall, HTC, PMTCT, and VMMC provider knowledge scores were low in all four countries. Within countries, there was some variation in scores across interventions, with provider knowledge scores for VMMC and PMTCT somewhat higher in Kenya than they were in Rwanda and Zambia (Fig 1). The distributions of scores for the three interventions in South Africa were similar. Across countries, provider knowledge scores for all three interventions were somewhat higher in Kenya and South Africa than they were in Rwanda and Zambia (Fig 2).

Table 4 shows the results of the GLM models. The coefficients are expressed as marginal effects. Model 1 estimated standard errors without clustering, Model 2 estimated robust standard errors, and Model 3 estimated standard errors clustering by facility. In Model 1, provider knowledge scores were higher among providers in facilities with senior staff members and among providers in facilities with higher proportions of intervention exclusive staff. We also found negative relationships between the outcome and provider years in position, average program age, provider working in another facility, average number of person-days of training, and management score. The robust standard errors (Model 2) were about double the size of the standard errors in Model 1, while in the model with clustering (Model 3) the standard errors were about three times the size of the errors in Model 1. In Model 3, only the coefficients for provider years in position, average program age, and management score remained statistically significant at conventional levels.

**Table 4:** Provider and facility characteristics associated with provider knowledge.

y = Knowledge score	Model 1		Model 2		Model 3	
	dy/dx	SE	dy/dx	SE	dy/dx	SE
Average patient load (weekly)	0.23	(0.18)	0.23	(0.41)	0.23	(0.57)
Senior staff	2.01***	(0.33)	2.01***	(0.76)	2.01	(1.52)
Provider years in position	-0.15***	(0.04)	-0.15*	(0.09)	-0.15*	(0.09)
Average program age (years)*	-1.42***	(0.17)	-1.42***	(0.37)	-1.42**	(0.62)
Provider works in another facility	-1.76**	(0.76)	-1.76	(1.58)	-1.76	(1.68)
Average percentage of exclusive staff for HIV*	1.44***	(0.20)	1.44***	(0.46)	1.44	(0.88)
Average number of person-days of training	-0.02**	(0.01)	-0.02	(0.02)	-0.02	(0.03)
Management score	-0.11***	(0.01)	-0.11***	(0.02)	-0.11***	(0.03)
Provider years of experience	0.06***	(0.02)	0.06	(0.06)	0.06	(0.07)
Provider years of education	0.52***	(0.06)	0.52***	(0.13)	0.52***	(0.16)
Provider age (years)	0.09***	(0.02)	0.09**	(0.05)	0.09	(0.07)
Doctors^a^	0.07	(0.66)	0.07	(1.48)	0.07	(1.90)
Counsellors^a^	-0.94**	(0.40)	-0.94	(0.91)	-0.94	(1.18)
PMTCT^b^	-0.81**	(0.39)	-0.81	(0.91)	-0.81	(0.94)
VMMC^b^	-1.60***	(0.46)	-1.60	(1.13)	-1.60	(1.25)
Private facilities	-0.87***	(0.34)	-0.87	(0.73)	-0.87	(1.40)
Hospitals	2.26***	(0.41)	2.26**	(0.94)	2.26	(1.80)
Rural facilities	2.64***	(0.34)	2.64***	(0.80)	2.64*	(1.54)
Total number of annual patients (thousands)*	-0.14***	(0.04)	-0.14	(0.10)	-0.14	(0.18)
Rwanda^c^	-6.90***	(0.70)	-6.90***	(1.46)	-6.90***	(2.50)
South Africa^c^	-2.38***	(0.62)	-2.38*	(1.32)	-2.38	(2.11)
Zambia^c^	-5.57***	(0.63)	-5.57***	(1.21)	-5.57***	(2.15)
Observations	1,796		1,796		1,796	

Notes: dy/dx columns represent marginal effects from Generalized Linear Models (GLM); standard errors in parentheses;

*** p<0.01,

** p<0.05,

* p<0.1;

HTC, HIV testing and counseling; PMTCT, prevention of mother-to-child transmission; VMMC, voluntary medical male circumcision;

^a^Reference category: Nurse;

^b^Reference category: HTC;

^c^Reference category: Kenya; models include dummies indicating observations that were imputed with the median (coefficients not reported);

Models 1, 2 and 3 include country-cadre and country-intervention interactions; Model 2 reports heteroskedasticity-robust standard errors; Model 3 reports cluster-robust standard errors (facility).

**Fig 3:**
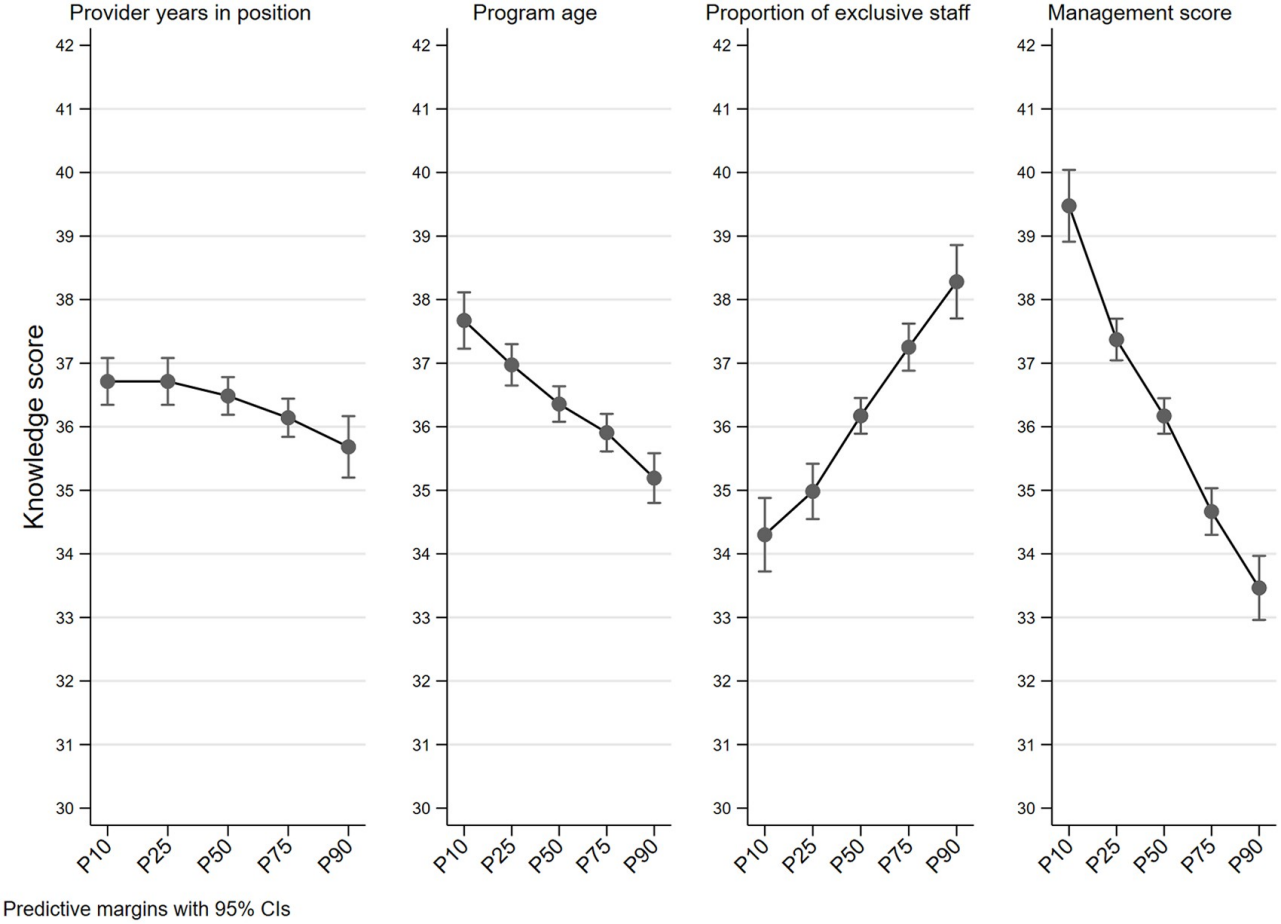
Margins plots of the provider knowledge score by percentiles of provider years in position, program age, proportion of exclusive staff, and management score.

Fig 3 shows the relationship between provider knowledge scores and provider years in position, program age, proportion of exclusive staff, and management score along percentiles of the distribution of these variables. Provider knowledge scores ranged from 40 to 33 (17.5% reduction) between p10 and p90 of the management score, from 38 to 35 (7.9% reduction) when shifting from p10 to p90 of program age, and from 37 to 36 (2.7% reduction) when moving between p10 and p90 of provider years in position. Knowledge scores ranged from 34 to 38 (11.7% increase) between p10 and p90 of the proportion of exclusive staff.

## Discussion

As others have argued, “the first step to closing the quality gap is to measure it [13].” As countries work to improve quality of HIV care and reach global HIV targets, measuring provider knowledge and other aspects of quality within individual countries and between countries is important. In this study of HIV service provider knowledge in Kenya, Rwanda, South Africa, and Zambia, we found mean knowledge scores of 36 for HTC, PMTCT, and VMMC. Within each country, some providers performed better, although few providers performed well. Between countries, knowledge scores were higher in Kenya and South Africa than they were in Rwanda and Zambia. The low scores we observed were consistent with prior studies using vignettes to assess provider knowledge in LMICs. Though we are unaware of previous work using vignettes to examine HIV service provider knowledge, this technique has been used to assess provider knowledge in an array of services including maternal and newborn health services [24–26, 28], childhood diarrhea and pneumonia [17–19, 21, 23, 26, 28], malaria [20, 21, 28], tuberculosis [17–19, 22, 26, 28, 64], and noncommunicable diseases [25, 27, 28]. Again and again, this growing body of work has shown that provider knowledge in resource-limited settings is low.

In our examination of the association between HTC, PMTCT, and VMMC provider knowledge and provider and facility characteristics, we observed differences in model results depending on whether we adjusted for facility-level clustering. This underscores the likely importance of organizational factors in individual HIV service provider knowledge. When we adjusted for intra-facility correlation, though the coefficients remained the same as in the models in which we did not adjust for clustering, many of the coefficients lost statistical significance. This suggests the presence of common, unobserved characteristics that affect the knowledge of providers belonging to the same cluster. This finding is in line with the arguments of organizational behavior researchers that knowledge accumulation occurs not just at the individual level, but at the organizational and industry levels as well [65]. Experts in organizational behavior argue that knowledge is a function of organizational experience [66], and that organizations learn through the accumulation of know-how and skill, improvements in workflow and coordination, and advances in technology [67]. Meanwhile, individuals embedded in organizations learn from knowledge reservoirs (e.g. treatment guidelines), tools, routines, social networks [38], and transactive memory systems [39, 40]. These transactive memory systems function like a computer or library for group members who can access information as part of the group that they might not access individually [68]. While these organizational characteristics are unobserved variables in our study, and therefore captured by the residual term of our regression models, our results are consistent with this literature. The potential of social learning and task coordination to increase provider knowledge should become part of future research agendas on service provision in resource-constrained settings.

Regardless of the regression specification model (either 1, 2, or 3), our results are consistent with five main hypotheses. First, individual experience is positively correlated with knowledge. Second, the presence of senior staff in a facility is positively correlated with knowledge. Third, there are diminishing returns of time on knowledge, both at the individual (provider years in position) and organizational (program age) levels. Fourth, staff dedication/specialization is positively associated with knowledge. Fifth, management and training are reactive rather than proactive with regard to improving provider knowledge.

Our finding that provider average patient load was positively associated with provider knowledge scores is consistent with the learning by doing literature that posits that as individuals repeat a task, they learn from direct experience and their knowledge about the task increases [38, 69]. Our hypothesis based on this literature was that more time spent on the job would translate into experience and knowledge as long as the workload was not so high that it overburdened staff. Previous assessments on the relationship between provider knowledge and workload and working time also found positive associations [22, 70].

Our result that provider knowledge scores were higher in facilities with at least one senior staff is supported by the extensive literature on champions as important facilitators in the successful implementation of health care services [71]. These individuals working in facilities with traits including enthusiasm, grit, persuasiveness, and intrinsic motivations and commitment to implementing improvements generally have a lot of influence [71, 72]. Champions tend to be more connected than other staff and this higher level of connectedness can facilitate a positive learning culture [72].

Our findings that provider years in position and average program age were negatively associated with provider knowledge are consistent with the assumption that knowledge capital depreciates over time and that knowledge can be unlearned or lost just as it can be learned or accumulated. Most literature on knowledge accumulation comes from manufacturing and finds sizeable rates of knowledge decay over time (e.g. [34, 73, 74]). Professional service workers like health care providers can be resistant to knowledge depreciation through formal education and training, compliance with standards and protocols, accreditation obligations, and continuous education requirements [75]. However knowledge decay can be considerable for procedural tasks which are dominant in health care [76, 77] and knowledge capital can depreciate when treatment guidelines evolve and take time to diffuse [35], staff do not have the motivation to master knowledge and skills [36], and facilities face staff turnover and organizational inertia [37].

Our findings on providers working in another facility and average proportion of staff in a facility focused exclusively on HTC, PMTCT, or VMMC are consistent with the literature on specialization and teamwork [39, 78, 79]. Providers working in another facility was negatively associated with provider knowledge, while average proportion of staff in a facility focused exclusively on HTC, PMTCT, or VMMC was positively associated with provider knowledge. Researchers have shown that specialization of the individuals that form a work group is an important dimension of the group’s transactive memory and that specialization is associated with more developed transactive memory systems [80, 81].

Our result that provider knowledge scores were higher in facilities with lower management scores was unexpected since several studies have found positive associations between quality of care and management practices [56, 57, 59, 82, 83]. It is true that these studies were conducted in high-resource settings and that there are low levels of adoption of modern management practices in low-resource settings, particularly in the public sector [6, 84]. It is also possible, however, that intensified management was a reaction to lower HIV service provider knowledge. There is evidence that management intensity increases when work processes are suboptimal and managers take actions to ensure that problems are addressed [85, 86]. This reactive feature of management will create a negative relationship when examining cross-sectional data like the ORPHEA study data explored here [87].

Our finding that number of person days of training in a facility was negatively associated with provider knowledge was also unexpected since training programs have been shown to improve provider practices when knowledge deficiencies impede the provision of quality services [88]. However, it is possible that, as with management practices, training of HIV service providers is reactive in our data and that training intensity increases when work processes are inferior.

Several limitations should be kept in mind when considering this analysis. Quality of care comprises multiple elements and our study focused on one dimension. Using vignettes, we measured the knowledge of health care providers. Vignettes measure what providers know and not what they do [18]. There is a growing literature describing the existence in LMICs of both a knowledge gap—a deficiency of knowledge or awareness of guidelines among providers [9, 18, 21, 25, 45] and a “know-do” gap—a gap between what providers know they should do and what they actually do [12, 16, 23, 64, 89–91]. Knowledge is a crucial starting point for quality of care, a necessary though not sufficient condition. Our knowledge scores were also limited in terms of the scope of the services studied, especially for PMTCT and VMMC. The PMTCT vignette focused on one antenatal care visit, while the VMMC vignette focused on pre- and post-operative counselling. We also note that there have been considerable changes in the ways in which HIV testing, prevention, and treatment interventions are implemented since the time of the study. However, our findings provide insights that are not specific to HTC, PMTCT, and VMMC, as implemented in 2012 and 2013. In this paper, we investigated aspects of provider knowledge that are relevant for quality of health care services beyond HIV services. The level of completeness of provider and facility characteristics in the ORPHEA surveys varied and limited our sample and the associations we could examine in this analysis. In addition, the ORPHEA study did not collect information on health systems and other environmental factors that may be associated with provider knowledge including country training and education schemes and health governance and regulations [13]. Our data were cross-sectional, and the results were descriptive. The potential endogeneity caused by reverse causality between provider knowledge and management practices and staff training days highlights the importance of longitudinal data to understand quality of care and its determinants.

## Conclusion

The policy recommendations arising from a cross-sectional study on the level of provider knowledge of HIV service providers and the associations between provider and facility characteristics and provider knowledge are limited [19]. Our cross-sectional data do not allow us to establish causality though our rich data are suitable to identify relevant hypotheses for future studies. Our exploration of expected provider knowledge at different percentiles of provider and facility characteristics does highlight the difficulty of raising provider knowledge scores from the low levels we observed. Our study also suggests that unobservable organizational factors at the facility level may facilitate communication, learning and knowledge. On the one hand, our study indicates that the presence of senior staff and staff dedication may enable knowledge acquisition. On the other hand, our study provides a note of caution on the potential knowledge depreciation correlated with the amount of time staff spend in a position and program age. Finally, our study underscores the importance of investing in human capital and improving managerial skills, with a focus on proactive management practices aimed at fostering a culture of reporting errors and continuous improvement. We advocate for gathering more granular and longitudinal data on the quality of HIV services and other health care services in sub-Saharan Africa.

## Supporting information

S1 Fig Proportion of correct answers to most difficult items for HTC, PMTCT, and VMMC by provider knowledge levels. Notes: HTC, HIV testing and counseling; PMTCT, prevention of mother-to-child transmission; VMMC, voluntary medical male circumcision.
(TIF)

S1 Table Description of HTC, PMTCT, and VMMC services examined in the ORPHEA study. Notes: HTC, HIV testing and counseling; PMTCT, prevention of mother-to-child transmission; VMMC, voluntary medical male circumcision; AZT, Zidovudine; 3TC, lamivudine; EFV, Efavirenz; FTC, Emcitricitabine; TDF, Tenofovir; sd, single dose; NVP, Nevirapine; CTX, Cotrimoxazole.
(DOCX)

S2 Table Average percent of items correctly identified by intervention, provider cadre, and by country. Notes: HTC, HIV testing and counseling; PMTCT, prevention of mother-to-child transmission; VMMC, voluntary medical male circumcision.
(DOCX)

S3 Table Management practice items by management domain. (DOCX)

S4 Table Description of variables. Notes: HTC, HIV testing and counseling; PMTCT, prevention of mother-to-child transmission; VMMC, voluntary medical male circumcision.
(DOCX)
