# Supplementary figures and images for: Characterizing health care provider knowledge: Evidence from HIV services in Kenya, Rwanda, South Africa, and Zambia
Source: PLoS One. 2021 Dec 2;16(12):e0260571. doi: 10.1371/journal.pone.0260571 (PMC8638969; doi:10.1371/journal.pone.0260571)

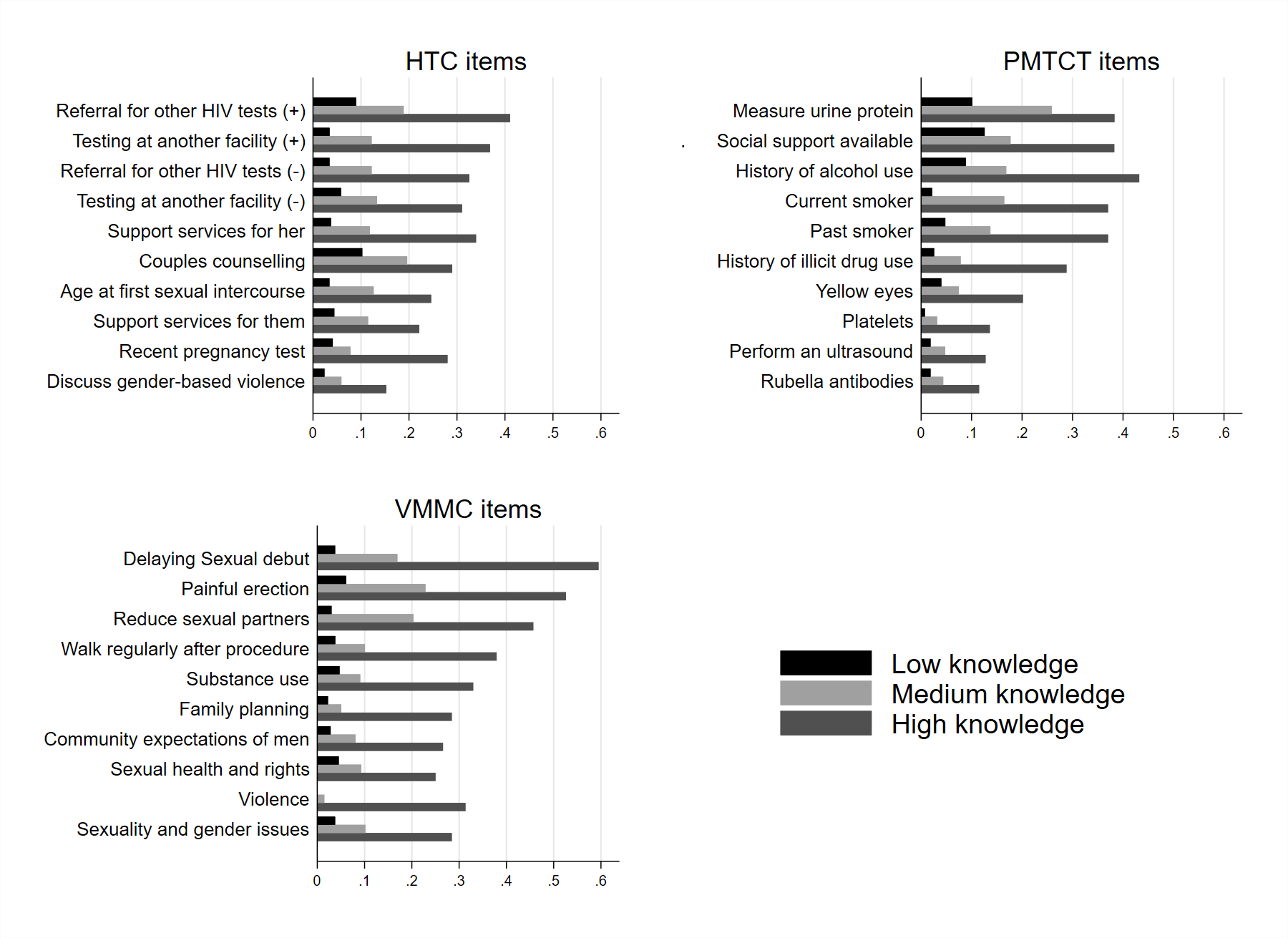

Supplement: S1 Fig — Notes: HTC, HIV testing and counseling; PMTCT, prevention of mother-to-child transmission; VMMC, voluntary medical male circumcision. (TIF) [file pone.0260571.s001.tif]
