# Supplementary material for: Characterizing health care provider knowledge: Evidence from HIV services in Kenya, Rwanda, South Africa, and Zambia
Source: PLoS One. 2021 Dec 2;16(12):e0260571. doi: 10.1371/journal.pone.0260571 (PMC8638969; doi:10.1371/journal.pone.0260571)
Supplement: S1 Table — Notes: HTC, HIV testing and counseling; PMTCT, prevention of mother-to-child transmission; VMMC, voluntary medical male circumcision; AZT, Zidovudine; 3TC, lamivudine; EFV, Efavirenz; FTC, Emcitricitabine; TDF, Tenofovir; sd, single dose; NVP, Nevirapine; CTX, Cotrimoxazole. (DOCX) [file pone.0260571.s002.docx]

| **Intervention** | **Description** |
| --- | --- |
| HTC | The HIV testing services assessed in the ORPHEA study were those implemented in sampled health facilities in Kenya, Rwanda, South Africa, and Zambia at the time of the study. The services encompassed a mix of client-initiated (where individuals actively seek HTC at a facility) and provider-initiated (where health care providers recommend HTC to individuals attending facilities as standard element of care) HTC. The HIV testing algorithms studied were those in place at the time of the study – all countries used serial rapid HIV test algorithms with laboratory ELISA used to resolve discordant results. |
| PMTCT | The prophylactic regimens considered in the ORPHEA study were those implemented at the time of the study. In Kenya, South Africa, and Zambia Option A was used, in Rwanda Options B and B+ were implemented. The drugs used in each country were as follows. In Kenya and Zambia, AZT was used during pregnancy and post-delivery AZT/3TC was administered to mothers for 7 days and NVP and CTX was given to infants. In Rwanda, TDF/3TC/EFV was administered to mothers and NVP and CTX was given to infants. In South Africa AZT was used during pregnancy and post-delivery sd TDF/FTC was administered to mothers and NVP and CTX was given to the infants. |
| VMMC | The VMMC services assessed in the ORPHEA study were routine VMMC services offered consistently year-round as well as intermittent services provided in facilities during high volume campaigns. The following VMMC features were assessed: VMMC counseling, HIV testing, medical examination, and surgical circumcision. While non-surgical devices (Pre-Pex and Shang Ring) were piloted in Rwanda at the time of the study, the vast majority of circumcisions in all countries were surgical. |
