# Supplementary material for: Characterizing health care provider knowledge: Evidence from HIV services in Kenya, Rwanda, South Africa, and Zambia
Source: PLoS One. 2021 Dec 2;16(12):e0260571. doi: 10.1371/journal.pone.0260571 (PMC8638969; doi:10.1371/journal.pone.0260571)
Supplement: S2 Table — Notes: HTC, HIV testing and counseling; PMTCT, prevention of mother-to-child transmission; VMMC, voluntary medical male circumcision. (DOCX) [file pone.0260571.s003.docx]

| **Intervention** | **Cadre** | **Kenya** | **Rwanda** | **South Africa** | **Zambia** |
| --- | --- | --- | --- | --- | --- |
| HTC | Physicians | 63.4 | 61.6 | 62.5 | 54.8 |
|  | Nurses | 62.4 | 49.8 | 63.8 | 52.6 |
|  | Counsellors | 60.7 | 47.8 | 64.9 | 53.3 |
| PMTCT | Physicians | 44.1 | 46.8 | 46.0 | 37.8 |
|  | Nurses | 45.7 | 37.7 | 42.5 | 41.2 |
|  | Counsellors | 43.6 | 36.0 | 26.7 | 39.0 |
| VMMC | Physicians | 39.9 | 26.4 | 35.7 | 35.0 |
|  | Nurses | 41.0 | 27.2 | 41.5 | 32.2 |
|  | Counsellors | 43.9 | 20.0 | 29.7 | 26.5 |
