# Supplementary material for: Characterizing health care provider knowledge: Evidence from HIV services in Kenya, Rwanda, South Africa, and Zambia
Source: PLoS One. 2021 Dec 2;16(12):e0260571. doi: 10.1371/journal.pone.0260571 (PMC8638969; doi:10.1371/journal.pone.0260571)
Supplement: S3 Table — (DOCX) [file pone.0260571.s004.docx]

| **Domains** | **Description** | **Questions** |
| --- | --- | --- |
| Operations management | This domain gathered information about the use of protocols in the facility, the use of human resources and the standardization of procedures | 1) How many total hours a day is this facility open on each of the days of the week and public holidays?  2) Funding based on amount of inputs used  3) Funding based on levels of drugs and supplies (i.e. no stock outs)  4) HIV patient register available and up-to-date HIV patient records  5) Facility has different financial reporting procedures  6) Performs task shifting  7) Supervisor did x (qualserv) during visit  8) Supervisor did x (audit) during visit  9) Supervisor did x (drugs) during visit  10) Supervisor did x (inqrecord) during visit  11) Supervisor did x (disimpl) during visit  12) Supervisor did x (meetstaff) during visit  13) Supervisor did x (meet_comm) during visit  14) Supervisor did x (other) during visit |
| Performance monitoring | This domain captured the review and tracking of provider performance, it measured whether continuous improvement processes were implemented and recorded | 1) Meetings to discuss management and administrative issues related to HIV/AIDS programs or services?  2) Frequency of meetings  3) Meeting report from last meeting  4) Governing board for facility  5) Funding based on quality of service  6) Information on levels of performance and quality of HIV prevention services routinely provided  7) Formal meetings organized regularly to inform this facility’s performance (e.g., productivity) and its quality of services regarding HIV prevention outcomes  8) Frequency performance of HIV/AIDS programs or services at this health facility assessed by this facility’s management |
| People management | This domain measured the availability of incentives and rewards for good performance, and the warnings and sanctions implemented for poor performance | 1) Staff at this facility receive rewards for high or improved performance  2) Staff ever receive extra payment/bonuses for good performance  3) Staff ever receive time off for good performance  4) Staff ever receive verbal recognition for good performance  5) Staff ever receive preferred rotation for good performance  6) Staff ever receive certificates for good performance  7) Staff ever receive training for good performance  8) Facility warn or apply sanctions to its staff for  absence without cause  9) Facility warn or apply sanctions to its staff for persistent tardiness  10) Facility warn or apply sanctions to its staff for persistent absenteeism  11) Facility warn or apply sanctions to its staff for impoliteness to patients  12) Facility warn or apply sanctions to its staff for impoliteness to other health workers  13) Facility warn or apply sanctions to its staff for impoliteness to supervisors  14) Facility warn or apply sanctions to its staff for persistent rudeness  15) Facility warn or apply sanctions to its staff for receiving many patient complaints  16) Theft or misappropriation  17) Last fiscal year, did any of the health workers at this facility receive work training  18) All medical personnel involved with HIV services at this facility receive training  19) All personnel (including medical personnel) involved with HIV services at this facility receive training |
| Community engagement | This domain measured the involvement of the community in facility decisions and activities and whether the community received information on facility performance | 1) Community representatives on governing board  2) Community advisory council  Community advisory council do in last year…  3) Buy items or award personnel bonuses from available funds  4) Communicate community complaints or any expressions of gratitude to the facility’s personnel  5) Monitor the delivery of HIV-related drugs to the facility  6) Monitor the delivery of HIV-related supplies to the facility  7) Participate in the discussions about priorities at the facility  8) Participate in the discussions about allocations for the various services  9) Facility inform the community about the performance (such as, productivity), politeness, and punctuality of HIV prevention services  10) Does the community have a role in monitoring and providing feedback to improve the performance  of the facility  11) Facility involve community groups in providing health services to the community |
