# Supplementary material for: Characterizing health care provider knowledge: Evidence from HIV services in Kenya, Rwanda, South Africa, and Zambia
Source: PLoS One. 2021 Dec 2;16(12):e0260571. doi: 10.1371/journal.pone.0260571 (PMC8638969; doi:10.1371/journal.pone.0260571)
Supplement: S4 Table — Notes: HTC, HIV testing and counseling; PMTCT, prevention of mother-to-child transmission; VMMC, voluntary medical male circumcision. (DOCX) [file pone.0260571.s005.docx]

| **Variables** | **Definition** | **Description** |
| --- | --- | --- |
| Country | Country in which the facility is located | Categorical variable that indicates in which of the 4 countries the facility is located: 1. Kenya 2. Rwanda 3. South Africa 4. Zambia |
| Intervention | Type of HIV prevention service: HIV testing and counselling (HTC); Prevention of mother to-child transmission (PMTCT); Voluntary Medical Male Circumcision (VMMC) | Categorical variable that indicates which of the 3 interventions the provider works on: 1. HTC 2. PMTCT 3. VMMC |
| Provider cadre | Provider cadre: Physician, Nurse & Counsellor | Categorical variable that indicates which of the 3 professional cadres the provider belongs to: 1. Physician (Medical officer, physician, clinical officer) 2. Nurse 3. Counsellor |
| Provider age (years) | Provider's age | Continuous variable. Provider's age in years at the time of the assessment |
| Provider years of education | Years of education completed by the provider | Continuous variable. Total years of education completed by the provider |
| Provider years in position | Time period in the current position at the facility | Continuous variable. Number of years since the provider started working in their current position |
| Provider years of experience | Working experience of the provider | Continuous variable. Number of years since the provider completed their studies |
| Average patient load (weekly) | Average number of patients seen by the provider per week | Continuous variable. Average number of patients seen by the provider in a week |
| Provider works in another facility | The provider works in another facility too | Dichotomous variable 0=No 1=Yes. It indicates if the provider works in another facility or not |
| Average % of exclusive staff for HIV | Average percentage of staff that is dedicated specifically to one intervention | Continuous variable. Average percentage of intervention exclusive staff in each clinic, expressed in standard deviations |
| Senior staff | Percentage of facilities with a senior staff | Continuous variable. The percentage of facilities with a senior staff member. We estimated a PCA score of seniority using the items: years of experience, years of education, years in the position, and provider's position. Then we estimated seniority score tertiles for each country. Providers in the highest tertile were considered senior staff |
| Average program age (years) | Average number of years interventions (HTC, PMTCT and/or VMMC) implemented in facility | Continuous variable. Average number of years interventions (HTC, PMTCT and/or VMMC) implemented in facility, expressed in standard deviations |
| Average number of person-days of training | Average number of person-days of training provided by facility to their staff | Continuous variable. The product of number of training days the facility offered to staff and the number of providers receiving training |
| Management score | Score based on management practices in facility | Continuous variable. Estimated PCA score of management that evaluates management practices implemented in the facility |
| Facility ownership | Ownership of facility | Dichotomous variable 0=public 1=private. It indicates if the facility is public or private |
| Facility type | Type of facility | Dichotomous variable 0=health clinic 1=hospital. It indicates if the facility is a health clinic or a hospital |
| Urbanicity | Urban/Rural classification of geographic area where facility is located | Dichotomous variable 0=Urban 1=Rural. It indicates if the facility is located in urban or rural area |
| Total number of annual patients (thousands) | Annual number of patients | Continuous variable. Annual number of patients receiving one or more of the three HIV prevention interventions during the costing years, in thousands |
| Knowledge score | Average score of provider knowledge | Continuous variable. Average score of provider knowledge, estimated for each intervention using a vignette instrument |
